# Pro-Calcific Environment Impairs Ischaemia-Driven Angiogenesis

**DOI:** 10.3390/ijms23063363

**Published:** 2022-03-20

**Authors:** Jocelyne Mulangala, Emma J. Akers, Emma L. Solly, Panashe M. Bamhare, Laura A. Wilsdon, Nathan K. P. Wong, Joanne T. M. Tan, Christina A. Bursill, Stephen J. Nicholls, Belinda A. Di Bartolo

**Affiliations:** 1 Discipline of Medicine, University of Adelaide, Adelaide, SA 5005, Australia; j.mulangala@centenary.org.au (J.M.); emma.akers@outlook.com (E.J.A.); emma.solly@sahmri.com (E.L.S.); joanne.tan@sahmri.com (J.T.M.T.); christina.bursill@sahmri.com (C.A.B.); 2 Vascular Research Centre, Heart Health Theme, South Australian Health and Medical Research Institute, Adelaide, SA 5000, Australia; panashe.bamhare@outlook.com (P.M.B.); wilsdonlaura@gmail.com (L.A.W.); nwon9940@alumni.sydney.edu.au (N.K.P.W.); 3 Faculty of Medicine and Health, School of Medicine, The University of Sydney, Sydney, NSW 2006, Australia; 4 Monash Cardiovascular Research Centre, Victorian Heart Institute, Monash University, Melbourne, VIC 3168, Australia; stephen.nicholls@monash.edu

**Keywords:** peripheral arterial disease, angiogenesis, vascular calcification, ischaemia

## Abstract

Peripheral arterial disease (PAD) is characterised by accelerated arterial calcification and impairment in angiogenesis. Studies implicate vascular calcification as a contributor to PAD, but the mechanisms remain unclear. We aimed to determine the effect of calcification on ischaemia-driven angiogenesis. Human coronary artery endothelial cells (ECs) were treated with calcification medium (CM: CaCl_2_ 2.7 mM, Na_2_PO_4_ 2.0 mM) for 24 h and exposed to normoxia (5% CO_2_) or hypoxia (1.2% O_2_; 5% CO_2_ balanced with N_2_). In normoxia, CM significantly inhibited tubule formation and migration and upregulated calcification markers of ALP, BMP2, and Runx2. CM elevated levels of calcification-protective gene OPG, demonstrating a compensatory mechanism by ECs. CM failed to induce pro-angiogenic regulators VEGFA and HIF-1α in hypoxia and further suppressed the phosphorylation of endothelial nitric oxide synthase (eNOS) that is essential for vascular function. In vivo, osteoprotegerin-deficient mice (OPG^−/−^), a calcification model, were subjected to hind-limb ischaemia (HLI) surgery. OPG^−/−^ mice displayed elevated serum alkaline phosphatase (ALP) activity compared to wild-type controls. OPG^−/−^ mice experienced striking reductions in blood-flow reperfusion in both 8-week-old and 6-month-old mice post-HLI. This coincided with significant impairment in tissue ischaemia and reduced limb function as assessed by clinical scoring (Tarlov). This study demonstrated for the first time that a pro-calcific environment is detrimental to ischaemia-driven angiogenesis. The degree of calcification in patients with PAD can often be a limiting factor with the use of standard therapies. These highly novel findings require further studies for full elucidation of the mechanisms involved and have implications for the development of therapies to suppress calcification in PAD.

## 1. Introduction

Peripheral arterial disease (PAD) refers to the obstruction or blockage of arteries of the lower extremities [1]. It is a progressive occlusive disease commonly present in an ageing population [2,3,4,5,6,7]. The pathogenesis of lower extremity PAD remains poorly described but is regarded secondary to atherosclerosis and constitutes a fatty plaque build-up within the arterial wall [8]. Despite the many clinical advances available to treat PAD, impairment in microcirculation continues to put patients at constant risk of ischaemia, increasing their risk of developing limb amputations which subsequently affect their quality of life [9]. Therapies for PAD are centred around alleviating stenosis and promoting tissue revascularisation following ischaemia [8], and while atherosclerosis is recognised as the main cause of PAD, acute or chronic limb ischaemia may be the result of various other cardiovascular risk factors [8]. Recent literature has implicated vascular calcification (VC) as a potential driver of PAD [8,9]. VC is a phenomenon of soft tissue calcification and is a major complication of atherosclerosis and is accelerated with ageing. It occurs due to the deposition of calcium and phosphate in the form of hydroxyapatite in the artery wall [8]. There are two primary forms of calcification: intimal calcification or medial calcification, both forms possessing different aetiologies [8].

Lower extremity calcification is commonly present in PAD and is highly associated with increased cardiovascular mortality and morbidity [10]. The most prominent type of calcification that occurs in the lower extremity is medial calcification, very distinct from intimal calcification occurring in the coronary arteries, although both may co-exist in diseases such as atherosclerosis [10]. Calcification in PAD is proposed to affect limb vascularisation due to reduced vessel compliance, increased arterial stiffening, and reduced coronary perfusion [10,11]. This suggests that the extent of calcification in lower extremity arteries may influence the severity of symptoms in PAD patients. The relationship between arterial calcification and PAD symptoms was previously reported to be independently associated with increased ischaemia in patients with PAD [10]. Because calcification affects limb vascularisation, it is thought to be a limiting factor for the treatment of PAD [8].

Angiogenesis is a fundamental process of the formation of new blood vessels from pre-existing vessels [7]. Endothelial cells play a key role in angiogenesis in which there are three main stages involving endothelial cell proliferation, migration, and tubule formation. In physiological settings, angiogenesis occurs postnatally and is involved in wound healing, repair, and growth and development [12]. It is also crucial for tissue revascularisation post-ischaemia, particularly following a myocardial infarction or stroke, as well as in peripheral vascular diseases. Angiogenesis is a well-regulated process driven by various growth factors including vascular endothelial growth factor (VEGFA) and hypoxia-inducible factor-1α (HIF-1α) [13].

Increasing calcium and phosphate levels within the normal range has been reported to positively corelate with cardiovascular disease conditions of hypercalcemia and hyperphosphatemia in chronic kidney disease (CKD) [14]. Currently, there lies a comprehensive body of literature describing calcification in a host of vascular beds and diseases; however, there is a paucity of information for its role relating to the peripheral arteries of the lower extremities. However, no studies have delineated the exact mechanisms linking vascular calcification in the regulation of angiogenesis. We hypothesised that a pro-calcific environment will be detrimental to endothelial-driven angiogenesis that will impact perfusion recovery in peripheral ischaemia. This paper sought to characterise the effect of calcification on functional angiogenesis in endothelial cells in vitro and investigate role of a calcification using the murine hind-limb model of angiogenesis in osteoprotegerin-deficient (OPG^−/−^) mice.

We report for the first time that a pro-calcific environment impairs endothelial angiogenic processes of migration and tubule formation and is primarily driven via the activation of several osteogenic signalling proteins of alkaline phosphatase (ALP), bone morphogenetic protein 2 (BMP2), Runx2, and receptor activator of nuclear kappa B ligand (RANKL) in vitro. Furthermore, in the hindlimb model of ischaemia-driven angiogenesis, we found that mice deficient in the calcification protective gene OPG experienced severely impaired blood flow reperfusion, impaired limb function, and increased tissue ischaemia with exacerbated foot necrosis. These studies highlight the devastating effects of a pro-calcific environment on ischaemia-driven angiogenesis and have implications for the development of therapies that can suppress calcification and subsequently help promote angiogenesis in PAD.

## 2. Results

### 2.1. Calcification Medium Upregulated Key Calcification Markers in ECs

**Figure 1:**
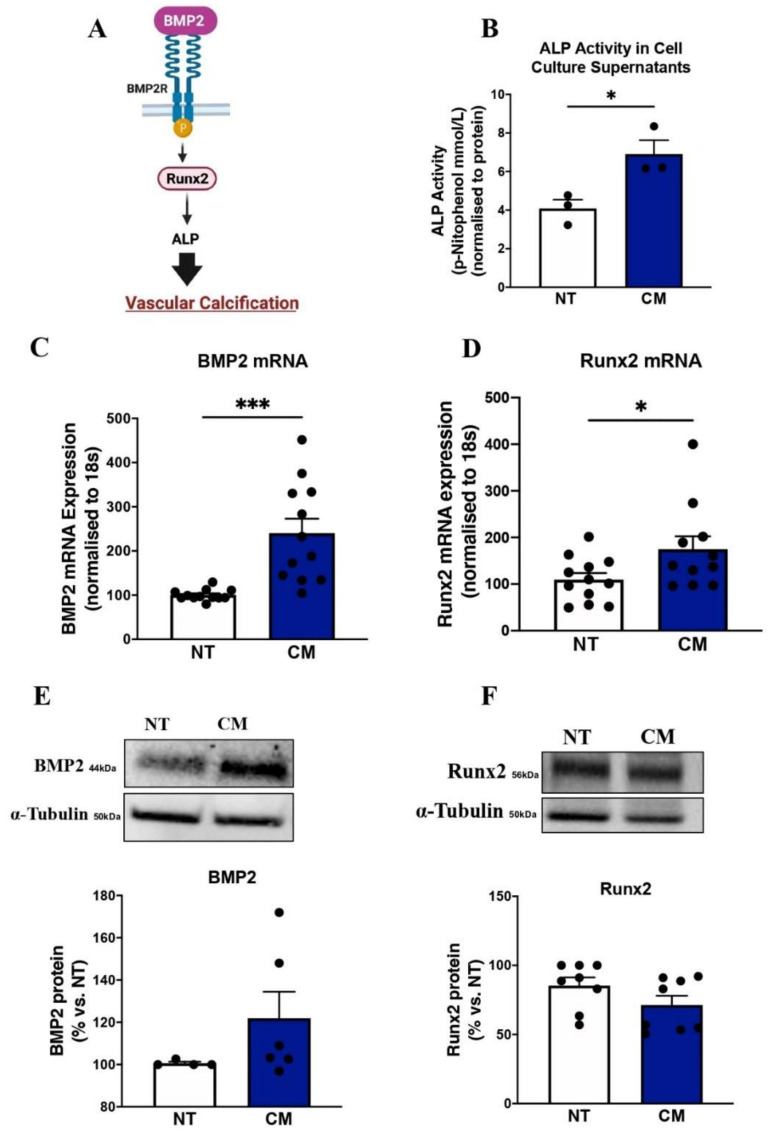
Calcification medium upregulated key calcification markers in ECs. HCAECs cultured and treated with calcification medium (CM) containing 2.0 mM HNa_2_PO_4_ and 2.7 mM CaCl_2_ for 24 h, in normoxia (37 °C humidified atmosphere with 5% CO_2_). RNA was extracted using Trizol, and RNA was reverse-transcribed into cDNA using iSCRIPT buffer. ALP assay (Wako, Lab Assay) was used to quantify ALP levels in cell culture supernatants following CM treatment. Real-time PCR was performed using universal SYBR^©^ Green Supermix. Relative changes in mRNA expression were calculated using the ∆∆Ct method. Whole-cell lysates were extracted to measure total and phosphorylated protein expression via immunoblotting technique. Even protein loading was confirmed by α-tubulin. (**A**) Molecular pathways for vascular calcification, (**B**) ALP activity, (**C**) BMP2 mRNA expression, (**D**) Runx2 mRNA expression, (**E**) BMP2 protein expression, (**F**) Runx2 protein expression. All data are represented as mean ± SEM; Student’s unpaired *t*-test, (*n* = 3/4), * *p* < 0.05, 0.01, *** *p* < 0.001. BMP2: bone morphogenetic protein 2, Runx2: runt-related transcription factor, ALP: alkaline phosphatase.

Firstly, we validated our calcification model by assessing calcification markers. The main transcription factor of vascular calcification (Runx2), potent osteoinductive factor BMP2, and the well-established bone-regulatory OPG-RANK-RANKL axis were assessed [15]. The signalling pathways for calcification induction involves binding of BMP2 to its receptor. The regulation vascular calcification is driven via two overlapping pathways. The first, BMP2 through the BMP receptor, resulted in phosphorylation and increase in transcription of the osteoblast transcription factor Runx2, which led to upregulation of Runx2-dependent calcification proteins such as ALP (Figure 1A). To determine the effects of a pro-calcific environment on endothelial cells, a calcification medium (CM) commonly used to stimulate calcification in vascular smooth muscle cells was utilised. CM increased alkaline phosphatase activity (measured in mmol/L) in ECs (CM: 6.90 ± 0.72 vs. control: 4.09 ± 0.45; *p* = 0.029) (Figure 1B). Furthermore, incubation with CM upregulated *Bmp2* mRNA levels (CM:240.40 ± 32.43 vs. control: 100.70 ± 3.66, *p* = 0.010; Figure 1C) and protein expression (CM:121.90 ± 12.52 vs. control: 100.70 ± 0.65, *p* = 0.211; Figure 1E), although it was not statistically significant. CM upregulated *Runx2* mRNA expression (CM:175. ± 27.72 vs. control: 109.80 ± 13.70, *p* = 0.042; Figure 1D), a response that was not translated at the protein level for Runx2 protein expression (*p* = 0.137; Figure 1F).

**Figure 2:**
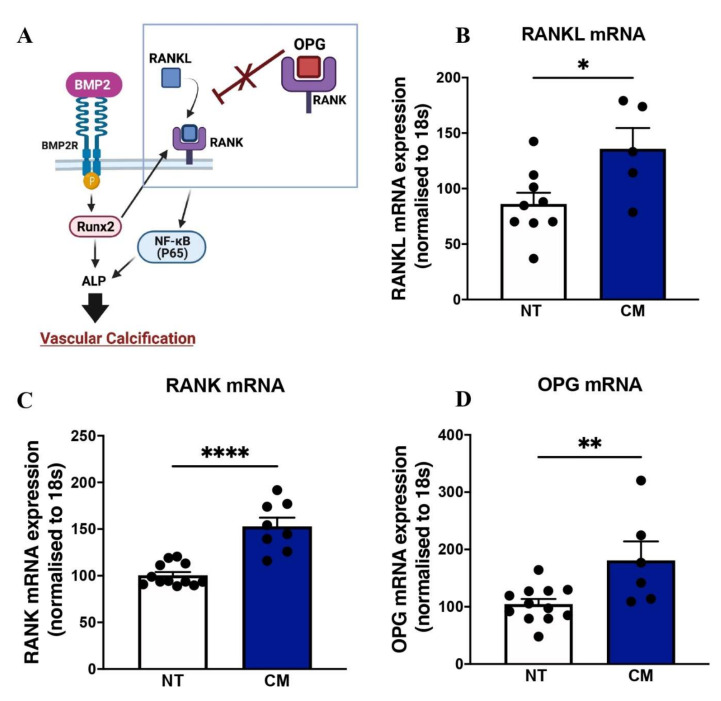
Calcification medium upregulated the OPG-RANK-RANKL pathway. HCAECs cultured and treated with calcification medium (CM) containing 2.0 mM HNa_2_PO_4_ and 2.7 mM CaCl_2_ for 24 h, in normoxia (37 °C humidified atmosphere with 5% CO_2_). RNA was extracted using Trizol, and RNA was reverse-transcribed into cDNA using iSCRIPT buffer. Real-time PCR was performed using universal SYBR^©^ Green Supermix. Relative changes in mRNA expression were calculated using the ∆∆Ct method. (**A**) Molecular pathway of vascular calcification, (**B**) RANKL mRNA expression, (**C**) RANK mRNA expression, (**D**) OPG mRNA expression. All data are represented as mean ± SEM; Student’s unpaired *t*-test, (*n* = 3/4), * *p* < 0.05, ** *p* < 0.01, **** *p* < 0.0001. OPG, osteoprotegerin, RANKL: receptor activator of nuclear factor ĸB ligand, RANK: receptor activator of nuclear factor ĸB, NF-κB (P65): nuclear factor κB (P65).

Analysis of the RANKL-RANK-OPG cytokine family showed upregulation of *Rankl* mRNA (CM: 135.80 ± 18.80 vs. control: 86.11 ± 10.08, *p* = 0.024; Figure 2B) and *Rank* mRNA (CM: 152.80 ± 9.34 vs. control: 100.60 ± 3.41, *p* < 0.0001; Figure 2C). Interestingly, incubation with CM upregulated *Opg* mRNA expression (CM: 181.10 ± 32.96 vs. control: 104.60 ± 8.94, *p* = 0.009; Figure 2D), which simply reflects a compensatory mechanism to counterbalance the increase in RANKL. Collectively, these data validate that our endothelial cells had an altered calcific response.

### 2.2. Calcification Medium Impaired Endothelial Cell Migration and Tubule Formation

**Figure 3:**
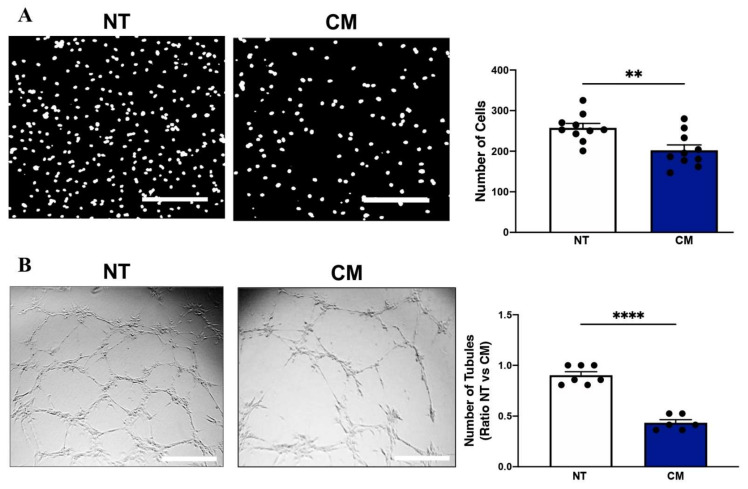
Calcification medium impaired endothelial cell migration and tubule formation. HCAECs cultured and treated with calcification medium (CM) containing 2.0 mM HNa_2_PO_4_ and 2.7 mM CaCl_2_ for 24 h, in normoxia (37 °C humidified atmosphere with 5% CO_2_). Functional assays were performed to assess angiogenesis. (**A**) Boyden cell migration assay, (**B**) Matrigel tubulogenesis assay. All data are represented as percentage of controls, mean ± SEM; Student’s unpaired *t*-test; (*n* = 3/7), ** *p* < 0.01, **** *p* < 0.0001. HCAEC: human coronary artery endothelial cells, M199: medium 199, NT: no treatment, CM: calcification medium.

Next, we wanted to assess the effect of CM on key EC angiogenic processes. Endothelial cells are key regulators of angiogenesis and play an important role following ischaemia. Proliferation, migration, and tubule formation are important processes in angiogenesis, and are strictly controlled by different extracellular signals [7,16,17]. Incubation of ECs with CM supressed EC migration (CM: 202.3 ± 13.37 vs. control: 257.6 ± 10.84, *p* = 0.048; Figure 3A). Consistent with these changes, CM further decreased EC tubule formation compared to untreated cells (CM: 0.98 ± 0.05 vs. control: 1.26 ± 0.06, *p* < 0.0001; Figure 3B). In keeping with the hypothesis, our pro-calcific environment had a detrimental effect on functional angiogenesis.

### 2.3. Calcification Medium Suppressed Angiogenic Signalling in Hypoxia

We further examined the underlying mechanisms of calcification on angiogenesis. In hypoxic conditions, the PI3K/Akt signalling pathway is activated, resulting in the induction of gene transcription of E3 ubiquitin ligases Siah 1 and Siah 2, which target and promote degradation of the propyl hydroxylase domains (PHDs), leading to HIF-1α stabilisation, and allowing HIF-1α translocation to the nucleus where it complexes with the HIF-1β subunit and binds to the hypoxia response element (HRE)^13^.

**Figure 4:**
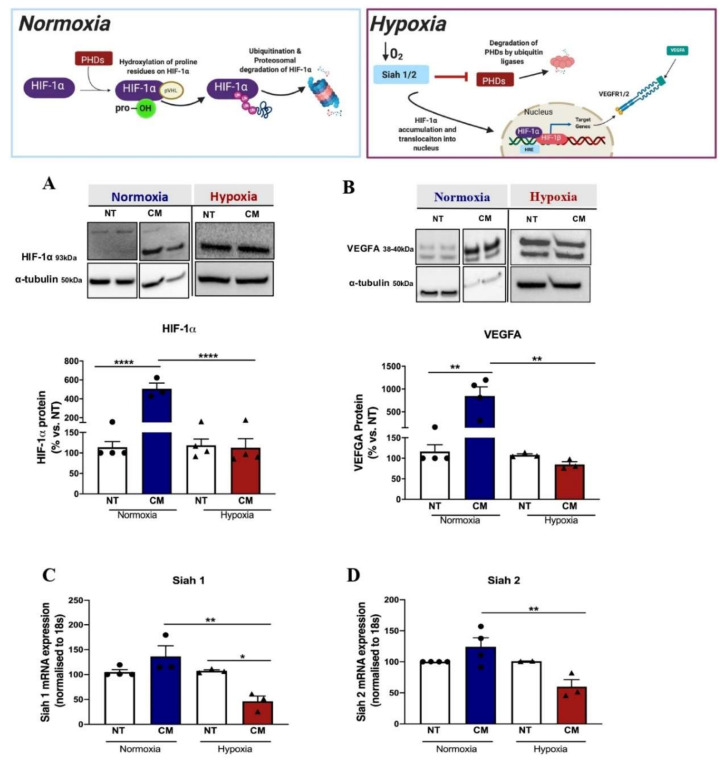
Calcification medium suppressed angiogenic signalling in hypoxia. HCAECs cultured and treated with calcification medium (CM) containing 2.0 mM HNa_2_PO_4_ and 2.7 mM CaCl_2_ for 24 h, in normoxia (37 °C humidified atmosphere with 5% CO_2_) and hypoxia (37 °C humidified atmosphere with 1.2% O_2_/5% CO_2_ balanced with N_2_) conditions. RNA was extracted using Trizol, and RNA was reverse-transcribed into cDNA using iSCRIPT buffer. Real-time PCR was performed using universal SYBR^©^ Green Supermix. Relative changes in mRNA expression were calculated using the ∆∆Ct method. mRNA was normalised to human 18 s. Whole cell lysates were extracted to measure total and phosphorylated protein expression via immunoblotting technique. Even protein loading was confirmed by α-tubulin. (**A**) HIF-1α protein expression, (**B**) VEGFA protein expression, (**C**) Siah 1 mRNA expression, (**D**) Siah 2 mRNA expression. All data are represented as mean ± SEM; unpaired Student’s *t*-test, * *p* < 0.05. All data are represented as percentage of controls, mean ± SEM; one-way ANOVA with multiple comparisons, Bonferroni test, (*n* = 3–4), * *p* < 0.05, ** *p* < 0.01, **** *p* < 0.0001. HCAEC, human coronary artery endothelial cells; M199, medium 199 (Sigma-Aldrich); HIF-1α, hypoxia-inducible factor-1 alpha; VEGF, vascular endothelial growth factor.

We found that CM had no effects on *Hif-1α* mRNA expression in normoxia (*p* < 0.999) or hypoxia (*p* = 0.579) (Figure S1A) but exhibited post-translational effects on HIF-1α expression and augmented its protein levels in normoxia compared to untreated cells (CM: 507.10 ± 59.70 vs. control: 113.80 ± 13.80, *p* < 0.001), with no effects observed in hypoxic conditions (*p* = 0.579, Figure 4A). Similarly, CM had no effect on *Vegfa* mRNA expression in the normoxia group (*p* = 0.082) compared to the hypoxia group (*p* = 0.285, Figure S1B); however, it demonstrated post-translational upregulation of VEGFA protein expression in normoxia (CM: 849.40 ± 197.60 vs. control: 116.50 ± 16.55, *p* = 0.004), with no effects seen in the hypoxia group (*p* > 0.999, Figure 4B). These data demonstrated that CM failed to induce both HIF-1α and VEGFA protein expression in response to hypoxia.

In addition, treatment of ECs with CM had no effect on the mRNA expression for *Siah 1* (*p* = 0.307) and *Siah 2* (*p* = 0.436) in normoxia. However, surprisingly, when ECs were stimulated with hypoxia, CM caused significant downregulation in *Siah1* mRNA expression compared to control cells (CM: 46.10 ± 10.62 vs. control: 107.40 ± 2.24, *p* = 0.020; Figure 4C) and *Siah 2* mRNA expression (CM: 59.99 ± 11.31 vs. control: 101.0 ± 0.30, *p* = 0.173; Figure 4D), although this was not statistically significant.

### 2.4. Calcification Medium Downregulated Downstream Signalling Pathways of eNOS

**Figure 5:**
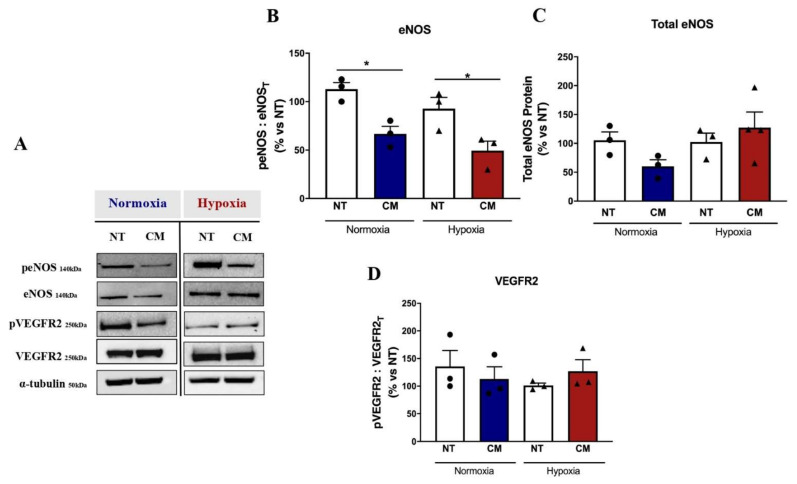
Calcification medium downregulated downstream signalling pathways of eNOS. HCAECs cultured and treated with calcification medium (CM) containing 2.0 mM HNa_2_PO_4_ and 2.7 mM CaCl_2_ for 24 h, in normoxia (37 °C humidified atmosphere with 5% CO_2_) and hypoxia (37 °C humidified atmosphere with 1.2% O_2_/5% CO_2_ balanced with N_2_) conditions. Whole-cell lysates were extracted to measure total and phosphorylated protein expression via immunoblotting technique. Even protein loading was confirmed by α-tubulin. (**A**) Representative images for phosphorylated eNOS (peENOS) and total eNOS (eNOS_T_), phosphorylated VEGFR2 (pVEGFR2) and total VEGFR2 (VEGFR2_T_), and α-tubulin (control); (**B**) total eNOS; (**C**) relative phosphorylated eNOS (peENOS) to total eNOS (eNOS_T_); (**D**) relative protein expression of phosphorylated VEGFR2 (pVEGFR2) to total VEGFR2 (VEGFR2_T_). All data are represented as mean ± SEM; one-way ANOVA with multiple comparisons, Bonferroni test, (*n* = 4), * *p* < 0.05. HCAEC, human coronary artery endothelial cells; VEGFR2, vascular endothelial growth factor receptor 2; eNOS, endothelial nitric oxide synthase.

To further determine the mechanism of calcification medium in driving angiogenesis, downstream signalling pathways were assessed. CM significantly mitigated eNOS phosphorylation in both normoxia (CM: 66.75 ± 7.86 vs. control: 112.9 ± 6.78, *p* = 0.015) and hypoxia (CM: 49.44 ± 9.78 vs. control: 92.75 ± 11.62, *p* = 0.020; Figure 5A,B), with no significant effects on total eNOS protein expression in either normoxia (*p* = 0.995) or hypoxia (*p* > 0.999; Figure 5A–C). In line with previous data from Figure 3A,B, where significant reductions in EC tubule formation and cell migration with CM were observed, this reduction in eNOS expression suggests a downstream signalling effect of calcification in supressing angiogenesis, despite increased angiogenic marker expression. CM also had no effect on VEGFR2 protein expression in both normoxia (*p* > 0.999) or hypoxia (*p* > 0.999; Figure 5A–D).

### 2.5. A Pro-Calcific Environment Adversely Impacted Ischaemia-Driven Revascularisation In Vivo

**Figure 6:**
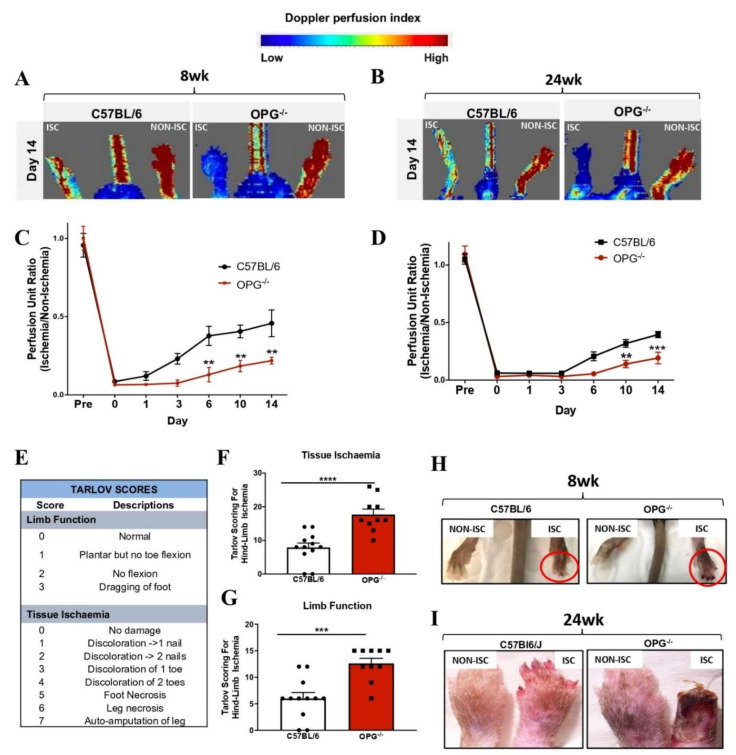
The pro-calcific environment adversely impacted ischaemia-driven revascularisation in vivo. Ischaemia-driven neovascularisation: femoral artery ligation was performed on 8-week-old and 6-month-old male C57BL/6 mice (controls) and OPG^−/−^ mice (*n* = 12/group). Blood flow perfusion was measured invasively using laser doppler perfusion imaging (LDPI). (**A**,**B**) Representative laser doppler perfusion images; images show high (red) to low (blue) blood flow at day 14. Perfusion unit ratio was determined on the basis of the ratio of ischemic (Isc) to non-ischemic (Non-Isc) hind-limb. Scale bars represent 50 μm. (**C**,**D**) Eight-week-old and aged OPG-deficient mice experienced impaired blood-flow reperfusion. (**E**) Tarlov scoring system used to assess limb function and tissue ischaemia. (**F**,**G**) OPG-deficient mice had increased tissue ischaemia and reduced limb function. (**H**,**I**) OPG-deficient mice developed necrotic toes at day 14 post-HLI surgery in 8-week-old and 24-week-old mice (*n* = 12/group). Data are represented as mean ± SEM, ** *p* < 0.01, *** *p* < 0.001, **** *p* < 0.0001. Statistical analysis was performed using a Mann–Whitney *t*-test and a two-way ANOVA with Bonferroni’s multiple comparison test. ALP, alkaline phosphatase; α-SMA, smooth muscle α-actin; OPG, OPG-deficient mice.

The osteoprotegerin knockout mouse is a well validated model used in calcification studies as it has been reported to exhibit extensive calcification in the aorta and renal arteries [18]. PAD is typically associated with ageing, and many HLI models use young adult mice, which is not often representative of the human condition [19]. We studied the effect of calcification on angiogenesis in both a young (8 week) and old model (24 week) to see if the duration of calcification may have an incremental effect on angiogenesis, as both PAD and calcification are associated with ageing. Laser doppler perfusion imaging showed striking reductions in blood flow reperfusion of 8-week-old OPG^−/−^ mice compared to wild-types at day 6 (*p* < 0.0001), day 10 (*p* < 0.01), and day 14 (*p* < 0.01) post-HLI (Figure 6A,C). Furthermore, 6-month-old OPG^−/−^ mice showed exacerbated effects on angiogenesis, and consistent with the younger cohort, had significant reductions in blood flow as measured by LDPI, particularly at day 10 (*p* < 0.01) and day 14 (*p* < 0.01, Figure 6B,D). We utilised clinical scoring to assess the functional changes in the limbs of both OPG deficient and wild-type mice post-HLI. Clinical scoring (Tarlov assessment) [20] revealed a significant increase in tissue ischaemia (177 vs. 95, *p* < 0.001; Figure 6E,F) and reduced limb function (126 vs. 72, *p* < 0.001; Figure 6E–G), with higher scores indicative of worse ischaemia and poor function, which does not occur in the absence of HLI. This was further associated with severe toe necrosis and self-amputations in the ischaemic limbs of these mice (Figure 6H,I) in both the young and old cohorts. Collectively, these results demonstrate that regardless of age, calcification adversely impacts ischaemia-driven revascularisation, resulting in dysregulated vascular response.

### 2.6. A Pro-Calcific Environment Suppressed Vascular Remodelling to Impair Ischaemia-Driven Angiogenesis

**Figure 7:**
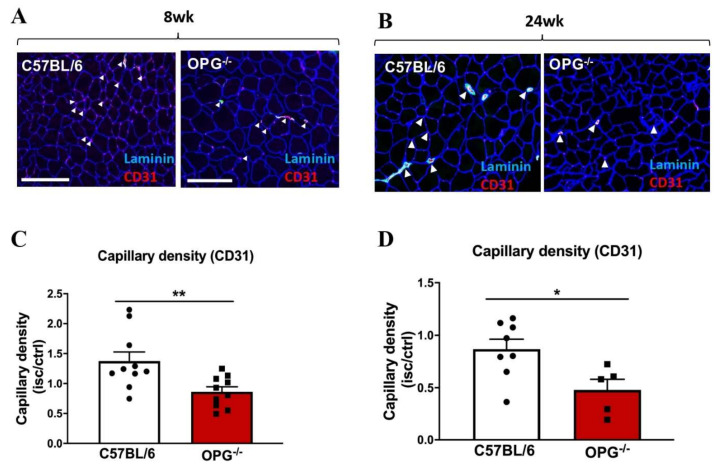
The pro-calcific environment suppressed vascular remodelling to impair ischaemia-driven angiogenesis. (**A**,**B**) Photomicrographs representing immunocytochemistry staining performed on OCT frozen gastrocnemius muscle sections to detect CD31^+^ neovessels (red staining), which was quantified as number of vessels (CD31^+^ per myocyte) using laminin staining (blue staining) (*n* = 6/group). White arrows indicate areas of the newly formed blood vessels. (**C**,**D**) OPG-deficient mice had significantly reduced capillary density compared to wild-type C57BL6/J mice. Data are represented as mean ± SEM, * *p* < 0.05, ** *p* < 0.01. Statistical analysis was performed using a Mann–Whitney *t*-test and a two-way ANOVA with Bonferroni’s multiple comparison test; OPG: OPG-deficient mice.

The inability for OPG-deficient mice to revascularise coincided with reductions in angiogenic vascular remodelling. Neovessel formation was significantly reduced in the gastrocnemius muscle of ischemic limbs in OPG^−/−^ mice, as early as 8 weeks, when stained for capillary density, as shown by CD31^+^ neovessels (0.80 ± 0.08 vs. 1.37 ± 0.15, *p* = 0.005; Figure 7A–C) compared to their wild-type controls. This result was also consistent at the later time point (*p* = 0.018; Figure 7B–D).

### 2.7. A Pro-Calcific Environment Differentially Regulated the Expression of Calcification and Angiogenic Markers in OPG^−/−^ Mice

**Figure 8:**
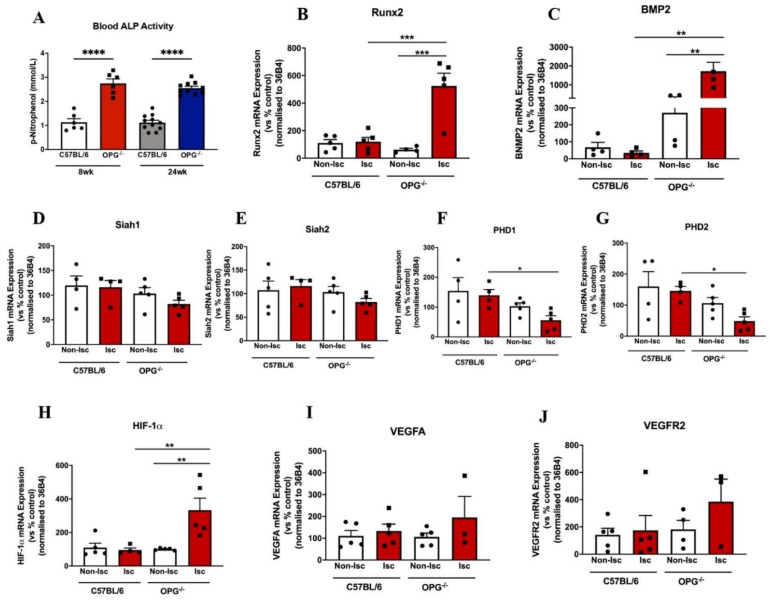
Pro-calcific environment differentially regulated gene expression of calcification and angiogenic genes in ischaemic limbs OF 8-week-old OPG^−/−^ mice. Femoral artery ligation was performed on 8-week-old male C57 BL/6 mice (controls) and OPG^−/−^ mice (*n* = 5/group). At day 14, mice were culled, and gastrocnemius tissues were harvested for RNA. Real-time PCR was performed to assess gene expression of key angiogenic markers in the gastrocnemius muscle of both C57 BL/6 and OPG^−/−^ mice. mRNA data are represented as non- ischemic (Non-Isc) for control hindlimb, and ischemic (Isc) for surgical hindlimb. (**A**) Blood ALP levels, (**B**) Runx2, (**C**) BMP2, (**D**) Siah1, (**E**) Siah2, (**F**) PHD1 mRNA expression, (**G**) PHD2, (**H**) HIF-1α (**I**) VEGFA, (**J**) VEGFR2. All data are represented as percentage of controls, mean ± SEM; * *p* < 0.05, ** *p* < 0.01, *** *p*<0.001, **** *p* < 0.0001, unpaired *t*-test, (*n* = 3–5). HIF-1α, hypoxia-inducible factor-1 alpha; VEGF, vascular endothelial growth factor; VEGFR2, vascular endothelial growth factor receptor 2; PHD1/2, propyl hydroxylase domains 1/2; Runx2: runt related transcription factor; BMP2: bone morphogenetic protein 2; OPG: osteoprotegerin.

**Figure 9:**
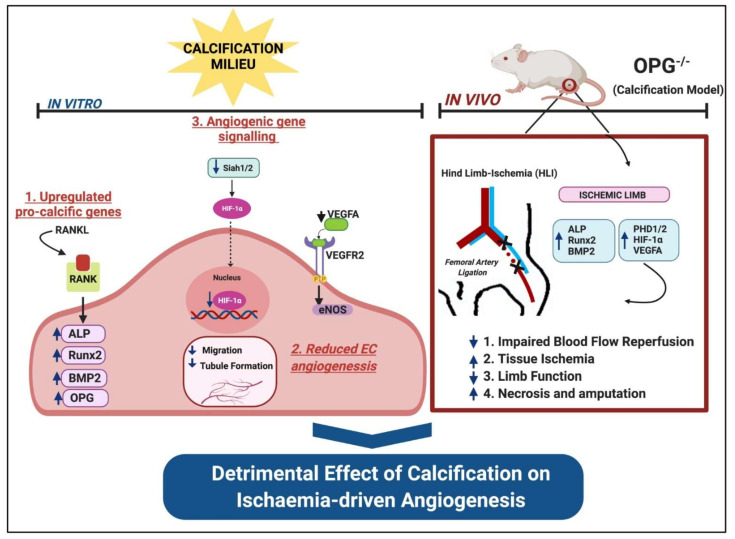
Summary schematic illustrating the effects of calcification on ischaemia-driven angiogenesis. Runx2: runt-related transcription factor, BMP2: bone morphogenetic protein, OPG: osteoprotegerin, ALP: alkaline phosphatase, PHD1/2: propyl hydroxylase domains 1/2, HIF-1*α*: hypoxia-inducible factor-1 alpha, VEGFA: vascular endothelial growth factor, VEGFR2: vascular endothelial growth factor receptor 2, RANKL: receptor activator of nuclear factor ĸB ligand, RANK: receptor activator of nuclear factor κB, eNOS: endothelial nitric oxide synthase, HLI: hind-limb ischaemia.

Calcification markers were assessed in the ischemic tissues of both control and OPG mice. OPG^−/−^ mice had elevated ALP activity (2.80 ± 0.18 vs. 1.14 ± 0.15, *p* < 0.001; Figure 8A) compared to C57BL/6 mice. OPG^−/−^ mice showed significant upregulation of *Runx2* mRNA expression compared to the ischemic limbs of the C57BL/6 controls (525.80 ± 91.40 vs. 120.00 ± 32.32, *p* < 0.01; Figure 8B), and this was also reflected in *Bmp2* mRNA levels (1722 ± 470.80 vs. 271.40 ± 103.40, *p* < 0.01; Figure 8C). Furthermore, when comparing non-ischemic to ischemic tissue in OPG^−/−^ mice, both *Runx2* (525.80 ± 91.40 vs. 62.04 ± 10.37, *p* < 0.0002; Figure 8B) and *Bmp2* (1722 ± 470.80 vs. 271.40 ± 103.40, *p* < 0.006) mRNA expression was significantly upregulated (Figure 8C and Figure 9).

During hypoxia/ischaemia, Siah 1 and 2 supress PHDs (PHD 1–3) which render HIF-1α for ubiquitination and degradation [21]. In this study, a pro-calcific environment had no effects on *Siah1* and *Siah 2* mRNA expression in both the ischemic and non-ischemic limbs of OPG^−/−^ mice compared to C57BL6/J controls (Figure 7D–E). However, OPG^−/−^ mice decreased PHD’s activity in their ischaemic limbs compared to C57BL/6 controls, with reductions in mRNA expression for *Phd1* (55.82 ± 14.95 vs. 139.60 ± 19.52, *p* < 0.05; Figure 8F) and *Phd2* (48.38 ± 13.73 vs. 146.1 ± 14.14, *p* < 0.01; Figure 8G). Interestingly, we observed significant upregulation of *Hif-1a* mRNA expression in the ischemic limbs of OPG^−/−^ mice compared to both the ischemic C57BL/6 (332.50 ± 72.05 vs. 94.75 ± 12.69, *p* < 0.01; Figure 8H) and its own non-ischemic tissue (332.5 ± 72.05 vs. 1100.2 ± 2.97, *p* < 0.05; Figure 8H and Figure 9).

## 3. Discussion

Vascular calcification (VC) is associated with atherosclerotic lesions and is thought to be a potential driver of PAD by affecting limb revascularisation following ischaemia [15]. In this case, angiogenesis or blood vessel formation is affected by the presence of calcific lesions within the peripheral arteries. However, there is still insufficient evidence regarding the exact mechanisms of calcification in regulating angiogenesis, particularly in ECs.

We report for the first time that a pro-calcific environment affected endothelial cell response to angiogenesis. Using a genetic knockout in vivo model of calcification, we found that deficiency in osteoprotegerin led to significantly impaired angiogenesis with reduced blood flow reperfusion and decreased neovessel formation in young mice, effects which were further exacerbated in the aged cohort (summarised in Figure 9). The OPG model used in this study provided a unique opportunity to assess the effects of elevated calcium on angiogenesis as these mice have been shown to develop extensive vascular calcification in their aorta and renal arteries [18]. While OPG-deficient mice result in greater serum calcium levels, and have been validated as a vascular calcification model, it cannot be discounted that the dramatic ischaemia-related effects could be due to the loss of OPG itself. OPG has been shown to have numerous functions in both physiological and pathological processes. Clinical studies have reported that higher levels of serum OPG is associated with poor cardiovascular outcomes in coronary artery disease, chronic kidney disease, and rheumatoid arthritis [22,23,24]. This therefore serves as a limitation in this study and could be explored as part of future experimental work.

There are several lines of evidence that bone-related osteogenic markers are involved in regulating vascular calcification in VSMCs, although this is lacking in the context of ECs. These regulators include members of the TNF superfamily of cytokines, receptor activator of nuclear factor ĸappa-B (RANK) and its ligand (RANKL); its soluble decoy receptor osteoprotegerin (OPG); and other bone regulatory factors such as runt related transcription factor 2 (runx2), the main transcriptional regulator of osteogenesis; bone morphogenetic protein 2 (BMP2); a strong inducer of bone formation; and ALP, an early marker of VC [25].

The mechanism underlying VC pathology suggests the differentiation of VSMCs towards an osteoblastic phenotype, as demonstrated in numerous in vitro studies [8,14,15]. On the basis of the evidence already available on these biomarkers of VC, as well as risk factors such as mineral imbalance, it is plausible that they are involved in regulating angiogenesis. The OPG/RANK/RANKL pathway has been previously implicated in the regulation of angiogenesis in ECs [26,27,28]. RANKL stimulates angiogenesis in vitro by acting as a chemostatic factor for ECs and inducing their migration and proliferation [28,29,30]. RANKL further stimulated a substantial increase in vascular permeability and promoted neoangiogenesis via induction of EC migration and proliferation in vivo [27,28,31]. In addition, VEGF can increase mRNA expression of RANK in human ECs, upregulating their angiogenic response to RANKL [27,28,32]. The upregulation of VEGFA in a pro-calcific environment coincided with increased expression of RANKL and RANK, further highlighting a relationship between calcification and angiogenic markers.

OPG is a soluble decoy receptor for RANKL and is a physiological and pharmacological inhibitor of bone resorption. It is highly elevated in cardiovascular disease, obesity, and diabetes [28,33,34]. The pro-angiogenic role of OPG has been well described to have effects on mature ECs by enhancing EC chemotaxis and cell survival [34,35] while promoting cell proliferation and vascular cord formation in Matrigel both in vitro and in vivo [36,37,38], therefore proposing its potential role in maintaining EC integrity [34]. Consistently, our studies show an increase in OPG expression and secretion from ECs in vitro after stimulation with calcification medium, such as elevations observed in disease states. Furthermore, OPG is expressed in VSMCs, and such increased expression in endothelial cells presumably reflects a vascular defence that helps to prevent excessive RANKL signalling via a negative feedback regulation and may thus be a compensatory mechanism for protection against disease [34,39]. Previous studies have shown that addition of RANKL to ECs reduced proliferation and induced apoptosis, suggesting RANKL to be an inhibitor of angiogenesis, and OPG a promotor of angiogenesis [38]. Our findings demonstrate that a pro-calcific environment is capable of upregulating both the drivers (ALP, Runx2, BMP2) and the inhibitor (OPG) of calcification in normoxia conditions. Furthermore, we see that CM had detrimental effects on cell migration and tubule formation, although increasing cell viability, all hallmark measures of angiogenesis. Previous studies have shown that in co-cultures of endothelial cells with osteoblasts, proliferation of ECs was enhanced by the production of osteoblast-derived angiogenic factors such as VEGFA [40]. This may therefore explain the increased migratory response of ECs with calcification medium, which coincides with increased VEGFA expression data both at the protein and mRNA levels.

Ischaemia is a potent stimulus for hypoxia-driven angiogenesis in pathological conditions of myocardial infarction, stroke, and peripheral arterial disease. In clinical settings, hypoxia increases hypoxia-inducible factor-1 alpha (HIF-1α), which further promotes VEGFA and VEGFR2 signalling to promote angiogenesis [12]. Interestingly, we demonstrated that this pro-calcific environment also promotes the release of key angiogenic markers HIF-1α and VEGFA, which may at least in part be a compensatory mechanism by the cells to counteract this mineral imbalance, thus favouring angiogenic protein synthesis. HIF-1α is expressed early in embryonic development and is necessary for bone development [41], while Runx2 is also expressed throughout skeletal development and is crucial for vascular invasion [42]. Deficiency in Runx2 is reported to result in vascularisation defects, mainly due to reduced VEGF expression, a key growth factor essential for angiogenesis [32,43]. Runx2 has also been shown to increase HIF-1α protein expression, mainly because it stabilises and protects HIF-1α from degradation, in both normoxic and hypoxic conditions [43]. Overexpression of Runx2 is also associated with significant increases in VEGF mRNA expression, and vice versa. This is thought to occur due to physical interactions of Runx2 and HIF-1α on the Runx2/RUNT domain [44] and possibly through binding of Runx2 on the VEGF promotor region [32]. In our results, suppression of angiogenesis was also associated with upregulation of angiogenic markers HIF-1α, VEGFA, and its receptor VEGFR2 in nonmovie, which corresponded with increased Runx2 mRNA levels. Since Runx2 is expressed in ECs of developing blood vessels [45], this may explain its role in regulating angiogenesis, highlighting the need for further studies to expand our understanding of this relationship. Additionally, the time of exposure of cells to hypoxia may influence the angiogenic response. Previous studies have reported that ECs exposed to 24 h of hypoxia attenuated VEGF-induced EC migration, proliferation, and tubule formation, demonstrating the negative effect of chronic hypoxia on angiogenesis [46]. In this study, cells were conditioned to hypoxia for only 4 h, reflecting acute hypoxia, potentially explaining the lack of expression of key angiogenic markers HIF-1α and VEGFA. On the basis of these results, we propose a potential mechanistic link in our study, wherein treatment of ECs with CM increased the expression of both Runx2 and HIF-1α. Therefore, it is clear that both factors can interact together to regulate VEGFA angiogenic signalling, which also showed increased expression with CM treatment.

In disease, ischemic tissue stimulates the growth of new blood vessels as an important strategy to revascularise the damaged area. The presence of calcification and a highly calcific environment has not been studied as a deterrent of this angiogenic process. Here, we used an OPG-deficient mouse model, as these mice develop extensive vascular calcification in their aorta and renal arteries [18]. Utilising this model, ischaemia-induced angiogenesis was assessed in vivo, demonstrating a profound detrimental suppression of vascularisation, with significant elevation of calcification markers (ALP, Runx2, and RANKL). Although we observed decreased revascularisation in the doppler data, we also observed significant upregulation in *Hif-1a* mRNA expression in the ischemic limbs of OPG^−/−^ mice compared to controls, a response that reflects a compensatory mechanism to increase angiogenesis. Indeed, OPG is known to be essential for angiogenesis in vitro [33]; however, to date, no studies have reported this critical observation. The pro-calcific milieu present in this mouse model is a unique opportunity to assess the effects of a pro-calcific environment and the resulting detrimental consequences.

There are other pathways to which endothelial cells can contribute to calcification that is independent of calcium phosphate supplementation. Currently, there is extensive evidence to demonstrate that the endothelium may contribute to calcification through a process known as endothelial mesenchymal transitions (EndMT), wherein the endothelial cells acquire an osteogenic phenotype and have increased level of expression for osteogenic characteristics and decreased expression in endothelial cell characteristics [47].

In addition, ageing plays a crucial role in endothelial dysfunction and the progression of calcification [48]. Our aged cohort of OPG-deficient mice displayed a significant loss of angiogenic ability in concert with extensive tissue ischaemia and impaired limb function, despite a small decrease in capillary and arteriole density. The combination of increased age and severe calcification in these mice created a largely unfavourable environment for revascularisation, similar to the consequences observed in patients with PAD. PAD patients presenting with femoral calcification often have reduced coronary perfusion and are at a higher risk for amputations [10,11]. Despite the clinical challenges, there is a lack of treatments and diagnostic tools to prevent or reverse cardiovascular calcification. However, there are a number of therapies that are under investigation for potential treatments for calcification; however, these therapies are often existing treatments for related conditions for osteoporosis therapies, CKD, and cardiovascular diseases. Bisphosphonates are pyrophosphate analogues used to treat osteoporosis but are also commonly used as targets for cardiovascular calcifications and have been shown to inhibit both vascular calcification and calciphylaxis in a several animal studies [49]. However, despite their effectiveness in animal models, there is still some debate over their efficacy for preventing vascular calcification in humans. Furthermore, bisphosphonates have anti-angiogenic properties and inhibit angiogenesis [50,51,52] and may therefore not be suitable treatment candidates for femoral artery calcifications in this case. OPG serves as a potential treatment for calcification due to their inhibitory effects, and administration of recombinant OPG has been shown to inhibit vascular calcification in animal studies [53,54].

It is important to note some limitations of the study with regard to the methodology for studying calcification. The most commonly used models for calcification are genetically modified mouse models, whereby medial calcification is induced by disrupting protective mechanisms by inhibiting calcification inhibitors. These knockout models include deficiency in MGP, Fetuin-A, OPN, and OPG. Furthermore, calcification can be induced by increasing hormonal regulation of phosphate, by affecting serum phosphate concentration. This method involves supplementation with vitamin D and is often used to study calcification in the setting of chronic kidney disease. This model, however, is not suitable to study angiogenesis, as vitamin D administration causes physical impairment and promotes weight loss. Furthermore, the majority of primary cell lines do not calcify spontaneously in vitro and require stimuli to induce calcification. Calcification medium is commonly supplemented with inorganic (sodium phosphate 1–5 mM) or organic (β-glycerophosphate, e.g., 10 mM) phosphate. Dexamethasone is a steroid that is often used to stimulate calcification in vitro. However, these readily available methods do not recapitulate hyperphosphatemia, hypercalcemia, inflammation, or a calcification environment as seen in the human body. Our study utilised the method of supplementing calcium and phosphate to cells as means to induce a physiological high calcific environment in these cells, a method that has been shown to calcify smooth muscle cells.

## 4. Materials and Methods

### 4.1. Cell Culture and Treatments

Human coronary artery endothelial cells (HCAEC) from Cell Applications (San Diego, CA, USA) were cultured in MesoEndo Cell Growth Media (212–500, Cell Applications). Cells were grown to 80% confluency then seeded at 1 × 10^5^ cells per well in 6-well plates. Cells were treated in Medium−199 (Sigma-Aldrich, St. Louis, MO, USA) containing 1.36 mM calcium which served as the no treatment (NT) control. Cells were also treated with calcium phosphate-conditioned medium containing 2.7 mM CaCl_2_ and 2.0 mM NaH_2_PO_4_ for 24 h and was denoted as calcification medium (CM). For the phosphorylation studies, cells were stimulated with recombinant human VEGF prior to harvesting. Cell culture media was changed every 2–3 days. Cell culture experiments were conducted under 2 different conditions of normoxia (5% CO_2_) for 24 h or hypoxia (1.2% O_2_ balanced with N_2_) for 4 h. All experiments were carried out in triplicate. All cells were used between passages 2 and 6.

### 4.2. Matrigel Tubulogenesis Assay

To assess tubulogenesis, growth factor-reduced Matrigel (356231; Corning Life Sciences, Corning, NY, USA) was carefully plated at 40 μL/well into a 96-well plate and left to polymerise at 37 °C for 30 min. Treated HCAECs were trypsinised, counted, and seeded onto the Matrigel at 1 × 10^4^ cells per well in 200 μL MesoEndo medium per well. Cells were then exposed to normoxic (5% CO_2_, 37 °C) and hypoxic conditions (1.2% O_2_ and 5% CO_2_ balanced with N_2_) for 4 h. HCAEC tubule formation was imaged at 10× magnification, and the number of tubes were counted and quantified in a minimum of 3 fields of view per well using FIJI is just ImageJ 1.52 h (National Institutes of Health, Bethesda, MA, USA).

### 4.3. Boyden Chamber Cell Migration Assay

Treated cells were harvested using Trypsin EDTA (15400054, ThermoFisher Scientific, Waltham, MA, USA) and resuspended in 500 μL of 3% FBS Opti-MEM medium (31985070, Life Technologies, Carlsbad, CA, USA) to make up cell suspension. A total of 600 μL of migration media (3% FBS Opti-MEM medium containing VEGF 10 ng/mL) was carefully added to the bottom chamber of a 24-well transwell. A total of 1 × 10^4^ cells were added into each transwell insert. Plates were incubated overnight at 37 °C. Transwell inserts were then removed, and cells on the upper surface of the transwell membrane were carefully scraped away using a cotton tip. Transwells were then rinsed in 1× PBS then fixed in 4% paraformaldehyde. Transwell membranes were cut out using a scalpel blade, and cells were mounted to the glass microscope slides with the cell-side up and stained with DAPI to detect nuclei using the Vectashield^®^ Antifade Mounting Medium (SK-5100, Vector Laboratories, Burlingame, CA, USA). Slides were imaged at 20× magnification in the dark at five separate fields at the same magnification with a fluorescent microscope. Analysis for cell counts were performed using FIJI is just ImageJ 1.52 h (National Institutes of Health, Bethesda, MA, USA).

### 4.4. Western Blotting

Treated cells were lysed using radioimmunoprecipitation assay (RIPA) lysis buffer for whole-cell lysates, containing protease and phosphatase inhibitors. Protein concentrations were determined using the Pierce Bicinchoninic (BCA) Protein Assay Kit, and total protein concentration between 10 and 20 μg of protein was loaded for Western blot analysis. Membranes were probed with antibodies for BMP2 (NBP119751, Novus Biologicals, Centennial, CO, USA, 1:1000), total HIF-1α (NB100–449, Novus Biologicals, 1:500), VEGFA (ab461154, Abcam, Trumpington, Cambridge, UK, 1:2000), total VEGFR2 (2479; Cell Signalling Technology, Danvers, MA, USA, 1:1000), phosphorylated (y^1175^) VEGFR2 (2478, Cell Signalling Technology, 1:1000), total eNOS (610297, BD Biosciences, San Jose, CA, USA, 1:1000), and phosphorylated (S^1177^) eNOS (61293, BD Biosciences, 1:1000). Even loading was confirmed by α-tubulin (ab729, Abcam, 1:5000) for total lysate. Protein levels were quantified and analysed using Bio-Rad ImageLab software (v.5.0, 170-9692, Bio-Rad Laboratories, Gladesville, CA, USA).

### 4.5. RNA Extraction, cDNA Synthesis, and Quantitative PCR (qPCR)

Total RNA was extracted using Trizol reagent (T9424, Sigma Aldrich) from endothelial cells and gastrocnemius tissues of mice following hind-limb ischaemia surgery. mRNA concentrations were quantified using the Nanodrop 2000 spectrophotometer (ThermoFisher Scientific, Waltham, MA, USA) and relative purity was determined using by the 260/280 ratio, with values between 1.8 and 2.0 deemed acceptable. A total of 100 ng/µL of total RNA was reverse-transcribed into cDNA using iScript buffer (Bio-Rad laboratories, Hercules, CA, USA) in the Bio-Rad T100™ Thermal Cycler. Real-time PCR was performed for the analysis of gene expression on endothelial cells and mouse tissues using the SsoAdvanced™ Universal SYBR© Green Supermix (1725275, Bio-Rad Laboratories), with 10 μmol of forward and reverse primers. The list of primers for each gene can be found in Tables S1 and S2. Relative changes in mRNA expression were calculated using the ∆∆Ct method, normalised to human 18 S and murine 36B4 genes.

### 4.6. Mouse Studies and Housing

All animal experimental procedures were conducted with approval from the South Australian Health and Medical Research Institute (SAHMRI) Animal Ethics Committee (SAM265) and were conducted in accordance with the Australian Code for the Care and Use of Animals for Scientific Purposes (8th edition, 2013). OPG^−/−^ mice (C57BL/6 background) were obtained from The Jackson Laboratory (Sacramento, CA, USA). Matching wild-type littermates (C57BL/6 background) were utilised as controls in all the experiments. Male mice were divided into two groups: 8 weeks of age for young adults, and 24 weeks of age for the ageing cohort. All animals were housed in the animal care facility at SAHMRI and maintained on a standard mouse chow diet (Speciality Feeds, Glen Forrest, WA, Australia).

### 4.7. Murine Hind-Limb Ischaemia (HLI) Model

The murine hindlimb ischaemia model (HLI) was performed as previously described [55] to ensure ischaemia of the distal regions of the hindlimb, using 8-week-old and 24-week-old male OPG^−/−^ mice and C57BL/6 wild-type littermate controls. Briefly, the proximal end of the left femoral artery and distal end of the saphenous vein were both ligated with 8-0 silk suture, and the artery and all side branches were dissected free and excised to induce ischaemia in the foot and the gastrocnemius muscle, and the overlying skin was sutured with 6-0 silk sutures (*n* = 12 per group). A sham operation was performed on the right hind-limb as a control.

### 4.8. Laser Doppler Perfusion Imaging (LDPI)

Blood flow reperfusion through the ischemic (left) and non-ischemic (right) limbs was determined using laser doppler perfusion imaging (LDPI) (moorLDI2-IR, Moor Instruments, Devon, UK) at baseline; immediately post-surgery; and on days 1, 3, 7, 10, and 14 following surgeries, with three scans taken per mouse at each time point.

### 4.9. Tarlov Scoring System

To assess tissue ischaemia and limb function, mice were assessed clinically after surgery using the well-established Tarlov clinical scoring system (modified), as previously described [20], at days 1, 3, 7, 10, and 14 post-surgery. For limb function, clinical scoring was scaled as 0 = normal gait movement, 1 = plantar but no toe flexion, 2 = no flexion, 3 = dragging foot. For tissue ischaemia, scoring was based on 0 = no damage, 1 = discoloration 1 nail, 2 = discoloration of 2 nails, 3 = discoloration of 1 toe, 4 = discoloration of 2 toes, 5 = foot necrosis, 6 = leg necrosis, and 7 = auto-amputation of leg.

### 4.10. Tissue and Blood Processing

Mice were anesthetised using isoflurane and euthanised by cardiac exsanguination 14 days post-surgery. Angiogenesis predominates in the ischemic distal bed [56], and thus the gastrocnemius muscles were isolated. Plasma was isolated for detection of calcium levels (701220, Calcium Assay, Cayman Chemical, Ann Arbor, MI, USA) and alkaline phosphatase activity (291–58601, LabAssay ALP, WAKO Chemicals, Richmond, VA, USA).

### 4.11. Immunohistochemistry

OCT-embedded gastrocnemius muscle tissues were sectioned to 5 μm thickness on the Shandon Cryotome E Cryostat (ThermoFisher Scientific) and mounted on superfrost plus^TM^ Adhesion Microscope Slides (J1800AMNT, ThermoFisher Scientific). Prior to staining, slides were dried overnight at room temperature, fixed in 2% paraformaldehyde for 10 min, then washed with 0.1 M glycine in PBS for 3 × 2 min to quench unreacted aldehydes. Tissues were washed in PBST containing 0.1% (*v*/*v*) Tween-20 for 3 × 5 min, followed by blocking in 5% goat serum (G9023, Sigma-Aldrich) in PBST for 1 h at room temperature.

Tissue sections were then incubated overnight with primary antibodies in antibody diluent to detect smooth muscle α-actin positive arterioles (1:1000, F3777, Sigma-Aldrich, St Louis, MO, USA) and laminin (1:200; MAB1905, Merck Millipore, Burlington, MA, USA) to assess myocytes. The next day following primary antibody incubation, tissues were washed with PBST for 4 × 2 min and were incubated with secondary antibodies Alexa FluorÒ 555 (1:1000; A-21434; Invitrogen, Carlsbad, CA, USA) in antibody diluent for 1 h at 37 degrees, which was followed by 6 × 2 min washes in PBST. Sections were again blocked with 5% goat serum for 1 h at room temperature and stained for CD31-positive neovessels (1:50, ab28364, abcam, Cambridge, UK). Slides were washed for 4 × 2 min in PBST then incubated with Alexa FluorÒ 647 (1:500; A-32733; Life Technologies, Carlsbad, CA, USA) secondary antibody in antibody diluent for 1 h. Slides were finally washed in PBST for 6 × 2 min each and mounted with Fluorescence Mounting Media (S302380–2, Agilent Technologies, Santa Clara, CA, USA), and a cover slip was applied to each slide. Control sections probed with only secondary antibodies were also run to assess and adjust for the degree of background staining during the imaging process. These were mounted with Prolong Antifade Mounting Media with DAPI (P36935, Life Technologies) so that cell nuclei could be detected.

All slides were allowed to dry in the dark for 24 h and were imaged on the Nikon Eclipse Ni-U microscope using NIS-Elements software, Ver51.03 (Melville, NY, USA) with five high-magnification fields (20×) of view taken for each section using the Cy5 (CD31), FITC (α-SMA), and Cy5 (laminin) filters. For analysis, CD31 + neovessels SMA + arterioles and myocytes were manually quantified using FIJI is just ImageJ software 1.52 h (National Institutes of Health, Bethesda, MA, USA).

### 4.12. Statistical Analysis

Analyses were performed using PRISM software (GraphPad Prism Version 7, San Diego, CA, USA). All data were analysed for statistical significance by an unpaired *t*-test amongst the independent groups of data. A value of *p* < 0.05 was deemed significant. One-way ANOVA test was also conducted, and using Bonferroni’s multiple comparison test, all treatment groups were compared to control groups. All data were represented as mean ± SEM of triplicates from 3 separately performed experiments.

## 5. Conclusions

This study provides important insights into the mechanisms by which calcification may regulate angiogenesis. While the mechanisms underlying the effects of calcification on angiogenesis are still not clear, this study has provided some evidence to identify how the calcification milieu has devastating effects on revascularisation (Figure 8). The degree of calcification in patients with PAD can often be a limiting factor when providing standard therapies. Our findings highlight the fact that to treat calcific PAD, we need to find new ways to recover angiogenesis following ischaemia.

## Data Availability

The data presented in this study are available on request from the corresponding author.

## Supplementary Materials

The following supporting information can be downloaded at: https://www.mdpi.com/article/10.3390/ijms23063363/s1.
