# Supplementary material for: Pro-Calcific Environment Impairs Ischaemia-Driven Angiogenesis
Source: Int J Mol Sci. 2022 Mar 20;23(6):3363. doi: 10.3390/ijms23063363 (PMC8954938; doi:10.3390/ijms23063363)
Supplement: Supplementary file 1 [file ijms-23-03363-s001.zip › ijms-1599267-supplementary.pdf]

Pro-Calcific Environment Impairs Ischaemia -Driven Angiogenesis

Jocelyne Mulangala [1,2], Emma J. Akers [1,2], Emma L. Solly [1,2], Panashe M. Bamhare [2], Laura A. Wilsdon [2], Nathan K. P. Wong [2,3], Joanne T. M. Tan [1,2], Christina A. Bursill [1,2], Stephen J. Nicholls [4] and Belinda A. Di Bartolo [3,*]

[1] Discipline of Medicine, University of Adelaide, Adelaide, SA, Australia, 5005; j.mulangala@centenary.org.au (J.M.); emma.akers@outlook.com (E.J.A); joanne.tan@sahmri.com (J.T.M.T.), christina.bursill@sahmri.com (C.A.B.)

[2] Vascular Research Centre, Heart Health Theme, South Australian Health and Medical Research Institute, Adelaide, SA, Australia, 5000; j.mulangala@centenary.org.au (J.M.); emma.solly@sahmri.com (E.L.S.); panashe.bamhare@outlook.com (P.M.B.); wilsdonlaura@gmail.com (L.A.W.); nwon9940@alumni.sydney.edu.au (N.K.P.W.)

[3] Faculty of Medicine and Health, The University of Sydney School of Medicine, Sydney, NSW, Australia, 2006; nwon9940@alumni.sydney.edu.au (N.K.P.W.), belinda.dibartolo@sydney.edu.au (B.A.D)

[4] Monash Cardiovascular Research Centre, Victorian Heart Institute, Monash University, Melbourne, Australia; stephen.nicholls@monash.edu

***** Correspondence: belinda.dibartolo@sydney.edu.au

SUPPLEMENTARY MATERIAL

Table S1: Human Primer sequences

	Human Primers		
	**Gene**	**Forward Primer 5'-3'**	**Reverse Primer 5'-3'**
**1**	***18S***	GAAGGCTGGGGCTCATTT	CAGGAGGCATTGCTGATGAT
**2**	***Rank***	ATGCGGTTTGCAGTTCTTGTC	ACTCCTTATCTCCACTTAGG
**3**	***Opg***	GCTTGAAACATAGGAGCTG	GTTTACTTTGGTGCCAGG
**4**	***Rankl***	ACTACCAGAAACGAGTGGGAA	GCATCTGTTCTCGGAAAACCT
**5**	***Runx2***	TGGTTACTGTCATGGCGGGTA	TCTCAGATCGTTGAACCTTGCTA
**6**	***Bmp2***	ACTACCAGAAACGAGTGGGAA	GCATCTGTTCTCGGAAAACCT
**7**	***Vegfa***	TGTGAATGCAGACCAAAGAAAGA	TGCTTTCTCCGCTCTGAGC
**8**	***Hif-1a***	AACGTCGAAAAGAAAAGTCTCG	CCTTATCAAGATGCGAACTCACA
**9**	***Siah1***	CACCAGCAGTTCTTCACCATTTAGC	CAATCGTCGCCTATGACCATTTAGC
**10**	***Siah2***	CCCTTCCTGCCTGCCCAGCCATCG	TCAGTGTCTATTAGCGCAATCG
**11**	***Vegfr2***	TTTGGTTCTGTCTTCCAAAGT	ATGCTCAGCAGGATGGCAA

Table S2: Murine Primer sequences

Murine Primers			
	**Gene**	**Forward Primer 5'-3'**	**Reverse Primer 5'-3'**
12	***36b4***	CAACGGCAGCATTTATAACCC	CCCATTGATGATGGAGTGTGG
13	***Vegfa***	GGCTGCTGTAACGATGAAG	CTCTCTATGTGCTGGCTTTG
14	***Vegfr2***	GCCCAGACTGTGTCCCGCAG	AGCGCAAGACCGGGGAGAGC
15	***Hif1- a***	TCCCTTGCTCTTTGTGGTTGGGT	AACGTAAGCGCTGACCCAGG
16	***Rankl***	CCAGCTATGATGGAAGGCTCA	ACCGAAGATAATGGACATGC
17	***Runx2***	AACGATCTGATTTGTGGCC	CCTGCGTGGGATTTCTTGGTT
18	***Bmp2***	GGGACCCGCTGTCTTCTAGT	TCAACTCAAATTCGCTGAGGAC
19	***Siah1***	GACTGCTACAGCATTACCCACT	GTTGGATGCAGTTGTGCCG
20	***Siah2***	CTAACGCCCAGCATCAGGAA	GAACAGCCCGTGGTAGCATA
21	***Phd1***	TAAGGTGCATGGCGGCCTGC	TGGCTGCTGCCCGTTCCTTG
22	***Phd2***	ATCACCTGGATCGAGGGCAA	CGTTCGGCCGTTTATCCTGT

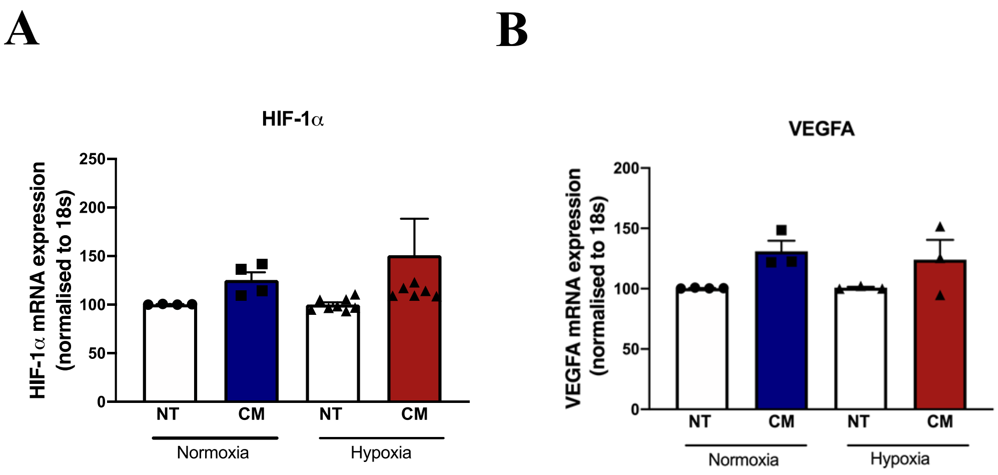

Figure S1: Pro-Calcific Environment Differentially Regulates Gene Expression of Calcification and Angiogenic Genes In ECs. HCAECs cultured and treated with calcification medium (CM) containing 2.0mM HNa_2PO_4 and 2.7mM $CaCl_2$ for 24h, in normoxia (37° C humidified atmosphere with 5% CO_2) and hypoxia (37° C humidified atmosphere with 1.2% O_2/5% CO_2 balanced with N_2) conditions. RNA extracted using Trizol, and. RNA was reverse transcribed into cDNA using iSCRIPT buffer. Real-time PCR was performed using universal SYBR© Green Supermix. Relative changes in mRNA expression were calculated using the ΔΔCt method. mRNA was normalised to human 18s. **(A)** HIF-1α mRNA expression, **(B)** VEGFA mRNA expression. All data is represented as percentage of controls, mean ± SEM; using a Mann-Whitney t-test and One-Way ANOVA with multiple comparisons, Bonferroni test; (n=6). VEGFA: vascular endothelial growth factor A, HIF-1α, hypoxia-inducible factor-1 alpha.

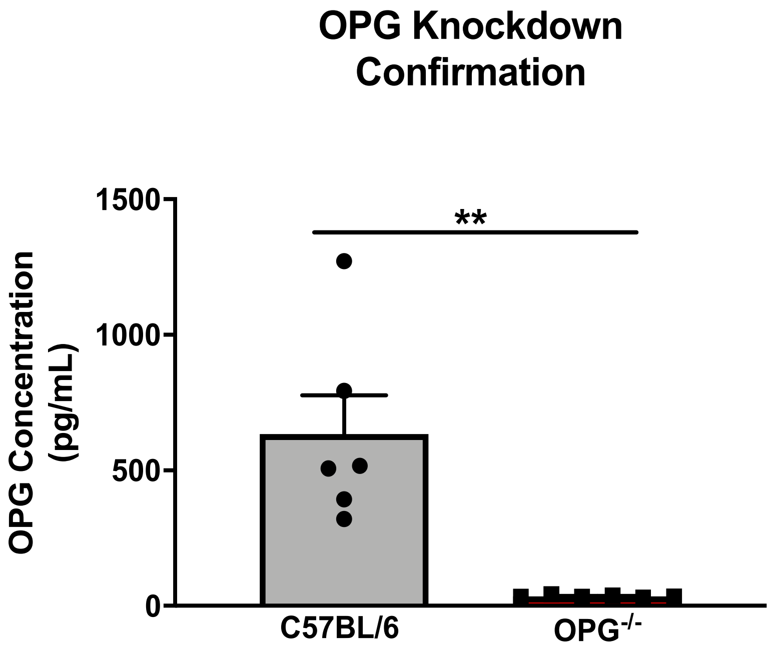

Figure S2: Confirmed Absence of OPG in OPG Deficient Mice. OPG knockdown was confirmed using OPG ELISA in 8wk old male C57BL/6 mice (controls) and $OPG^{-/-}$ mice. All data is represented as percentage of controls, mean ± SEM; using a Mann-Whitney t-test and One-Way ANOVA with multiple comparisons, Bonferroni test; (n=6),*P,0.002, OPG: OPG deficient mice.
